# Supplementary material for: The lineage stability and suppressive program of regulatory T cells require protein O-GlcNAcylation
Source: Nat Commun. 2019 Jan 21;10:354. doi: 10.1038/s41467-019-08300-3 (PMC6341091; doi:10.1038/s41467-019-08300-3)
Supplement: Supplementary file 1 — Supplementary Information [file 41467_2019_8300_MOESM1_ESM.pdf]

Liu et al.

The lineage stability and suppressive program of regulatory T cells require protein O-GlcNAcylation

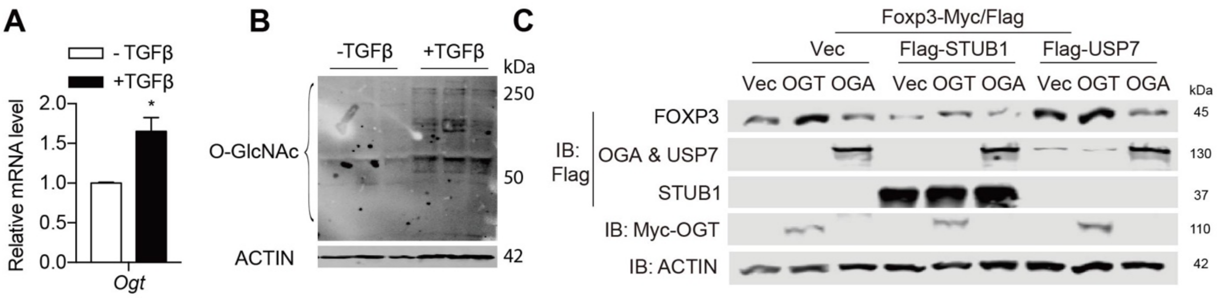

Supplementary Fig. 1 (A, B) $CD4^{+}CD25^{-}$ naïve T cells isolated from the LNs and spleen of wildtype mice were activated for 5-day with anti-CD3/CD28 beads in the presence of TGF-β to generate iTreg cells ex vivo. mRNA levels of *Ogt* (A, n = 3) and global protein O-GlcNAcylation (B) were measured. (C) Expression of FOXP3 protein in HEK 293 cells co-transfected with OGT or OGA and STUB1 or USP7. Data are shown as mean ± s.e.m. *, $p<0.05$ by unpaired student's t-test.

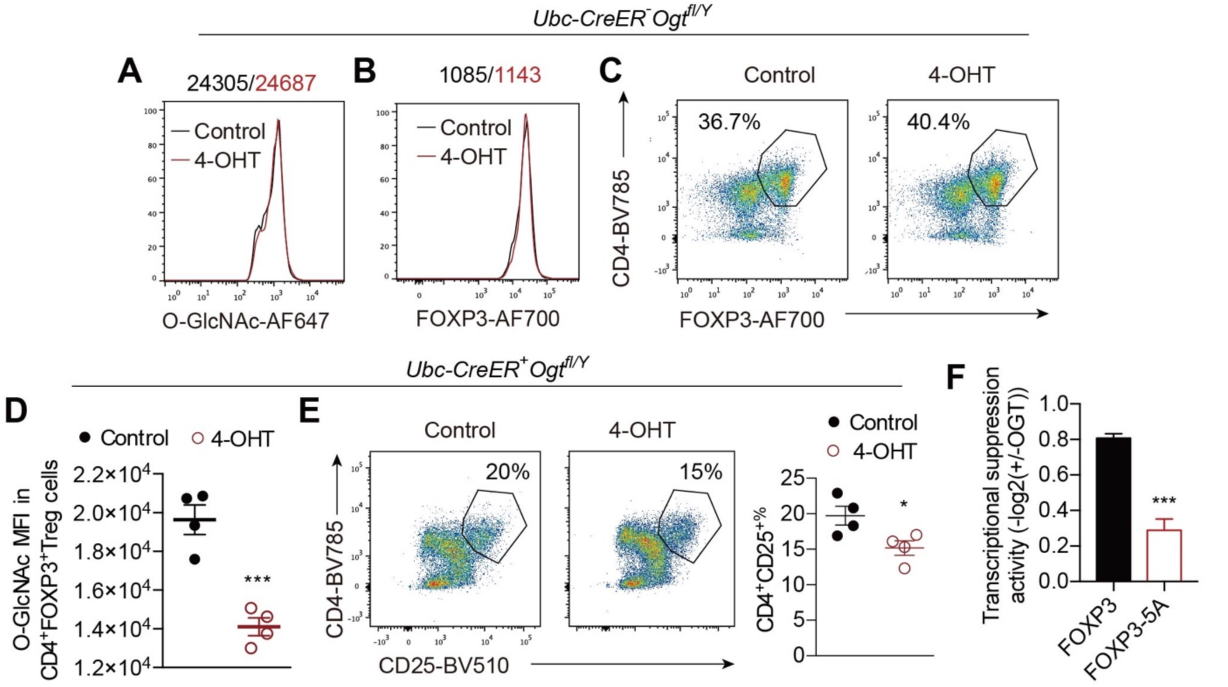

Supplementary Fig. 2 (A-C) Isolated Treg cells from *Ubc-CreER⁻Ogt*$^{fl/Y}$ mice were treated for 3-day 4-OHT ex vivo. Ethanol was used as a control. MFI of O-GlcNAcylation (A) and FOXP3 (B) among $CD4^+FOXP3^+$ Treg cells. Representative flow cytometry of $CD4^+FOXP3^+$ Treg cells were shown in (C). (D) MFI of O-GlcNAcylation in $CD4^+FOXP3^+$ Treg cells from *Ubc-CreER*$^+$*Ogt*$^{fl/Y}$ mice that were treated with ethanol or 4-OHT, n = 4. (E) Representative flow cytometry and quantification of the frequencies of $CD4^+CD25^+$ Treg cells from *Ubc-CreER*$^+$*Ogt*$^{fl/Y}$ mice that were treated with ethanol or 4-OHT, n = 4. (F) Forkhead responsive element (FHRE)-luciferase assay in HEK 293 cells transfected with FOXP3 or FOXP3-5A in the presence/absence of OGT. pIS1-Rluc was co-transfected to control transfection efficiency. OGT-induced activation of FOXP3 suppressive activity on FHRE was plotted (n = 6). Data are shown as mean ± s.e.m. *, $p<0.05$, ***, $p<0.001$ by unpaired student's t-test.

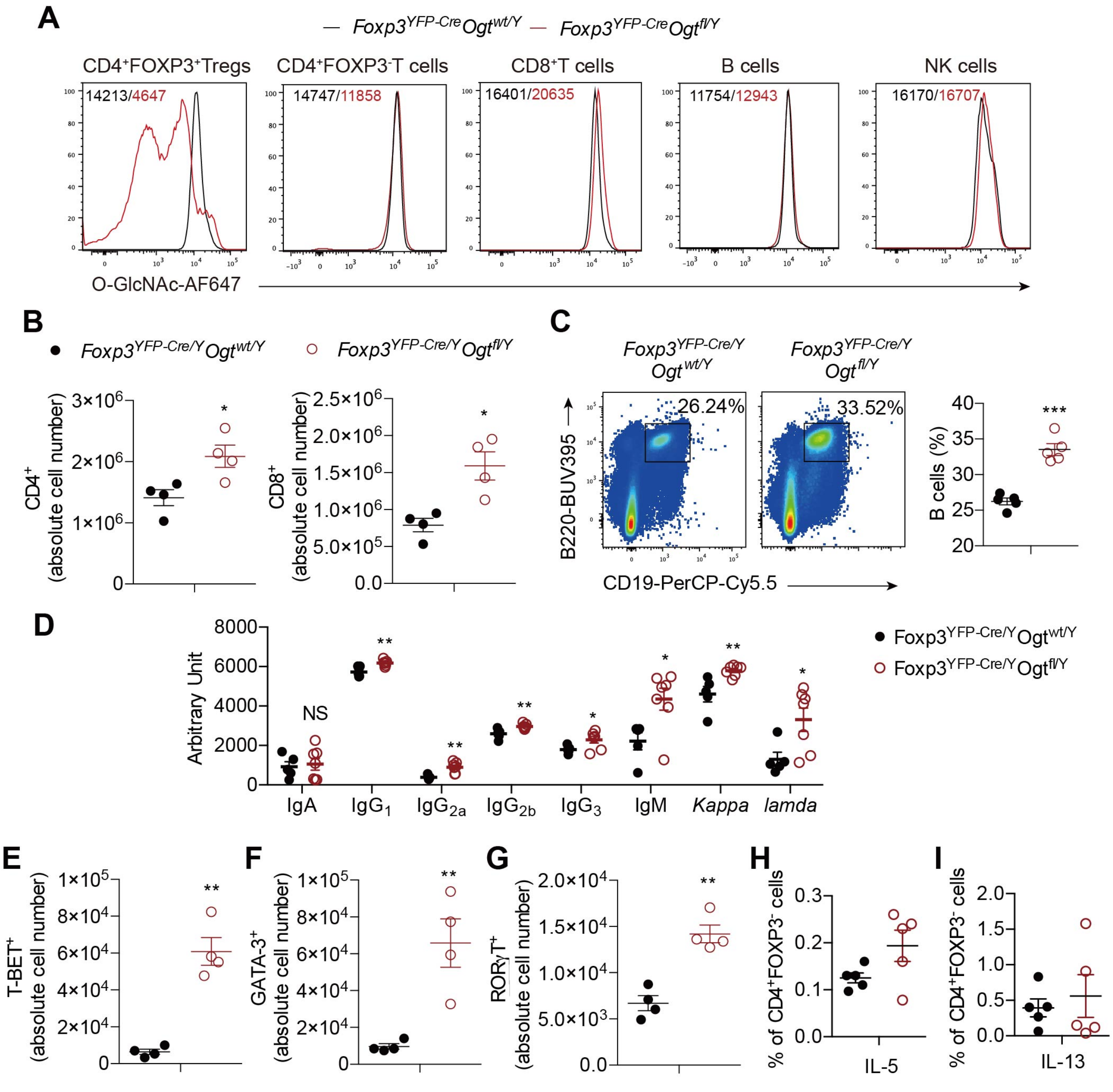

Supplementary Fig. 3 (A) MFI of O-GlcNAcylation in indicated cell populations from *Foxp3*$^{YFP\text{-}Cre/Y}$ *Ogt*$^{wt/Y}$ and *Foxp3*$^{YFP\text{-}Cre/Y}$ *Ogt*$^{fl/Y}$ mice. (B) Total number of $CD4^+$ and $CD8^+$ T cells in the LNs from 2-week-old *Foxp3*$^{YFP\text{-}Cre/Y}$ *Ogt*$^{wt/Y}$ and *Foxp3*$^{YFP\text{-}Cre/Y}$ *Ogt*$^{fl/Y}$ mice, n = 4 each group. (C) Representative flow cytometry plot and quantification of the frequencies showing $CD19^+B220^+$ B cells among single cells in the LNs of 2-week-old *Foxp3*$^{YFP\text{-}Cre/Y}$ *Ogt*$^{wt/Y}$ and *Foxp3*$^{YFP\text{-}Cre/Y}$ *Ogt*$^{fl/Y}$ mice (n = 5). (D) Levels of IgA, IgG_1, IgG_{2a}, IgG_{2b}, IgG_3, IgM, *Kappa* and *lamda* in sera of *Foxp3*$^{YFP\text{-}Cre/Y}$ *Ogt*$^{wt/Y}$ (n = 5) and *Foxp3*$^{YFP\text{-}Cre/Y}$ *Ogt*$^{fl/Y}$ (n = 7) mice. (E-G) Total numbers of T-BET^+ (E), GATA-3^+ (F) and $RORγT^+$ (G) cells among $CD4^+$ T cells in the LNs from 2-week-old *Foxp3*$^{YFP\text{-}Cre/Y}$ *Ogt*$^{wt/Y}$ and *Foxp3*$^{YFP\text{-}Cre/Y}$ *Ogt*$^{fl/Y}$ mice, n=4 each group. (H, I) Frequencies of IL-5^+ (H) and IL-13^+ (I) cells in $CD4^+FOXP3^-$ T cells stimulated with PMA/Ionomycin in the LNs of 2-week-old *Foxp3*$^{YFP\text{-}Cre/Y}$ *Ogt*$^{wt/Y}$ and *Foxp3*$^{YFP\text{-}Cre/Y}$ *Ogt*$^{fl/Y}$ mice, n = 5. Data are shown as mean ± s.e.m. *, p<0.05; **, p<0.01; ***, p<0.001 by unpaired student's *t*-test.

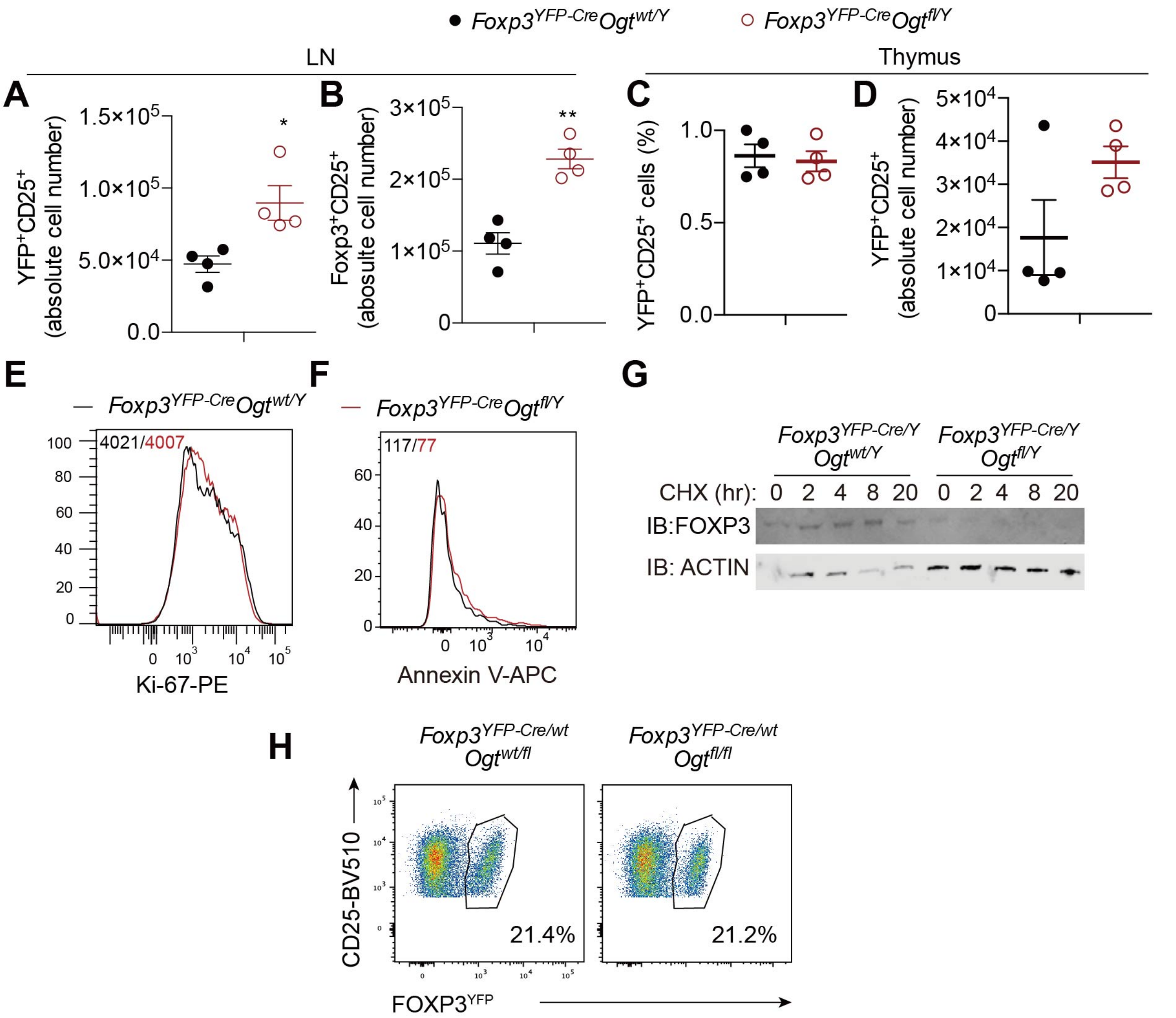

Supplementary Fig. 4 (A, B) Total numbers of YFP$^+$CD25$^+$ (A) and FOXP3$^+$CD25$^+$ (B) Treg cells among CD4$^+$ T cells in the LNs from 2-week-old *Foxp3$^{YFP\text{-}Cre/Y}$ Ogt$^{wt/Y}$* and *Foxp3$^{YFP\text{-}Cre/Y}$ Ogt$^{fl/Y}$* mice, n = 4 each group. (C, D) Frequency (C) and total number (D) of YFP$^+$CD25$^+$ Treg cells among CD4$^+$ T cells in the thymus from 2-week-old *Foxp3$^{YFP\text{-}Cre/Y}$ Ogt$^{wt/Y}$* and *Foxp3$^{YFP\text{-}Cre/Y}$ Ogt$^{fl/Y}$* mice, n = 4 each group. (E, F) MFI of Ki-67 (E) and Annexin V (F) among YFP$^+$CD25$^+$ Treg cells in the LNs from 2-week-old *Foxp3$^{YFP\text{-}Cre/Y}$ Ogt$^{wt/Y}$* and *Foxp3$^{YFP\text{-}Cre/Y}$ Ogt$^{fl/Y}$* mice. (G) FOXP3 stability in Treg cells isolated from *Foxp3$^{YFP\text{-}Cre/Y}$ Ogt$^{wt/Y}$* and *Foxp3$^{YFP\text{-}Cre/Y}$ Ogt$^{fl/Y}$* mice was determined by CHX treatment. (H) Frequencies of YFP$^+$ Treg cells in the LNs from *Foxp3$^{YFP\text{-}Cre/wt}$ Ogt$^{wt/fl}$* and *Foxp3$^{YFP\text{-}Cre/wt}$ Ogt$^{fl/fl}$* female mice. Data are shown as mean ± s.e.m. *, $p<0.05$; **, $p<0.01$ by unpaired student's *t*-test.

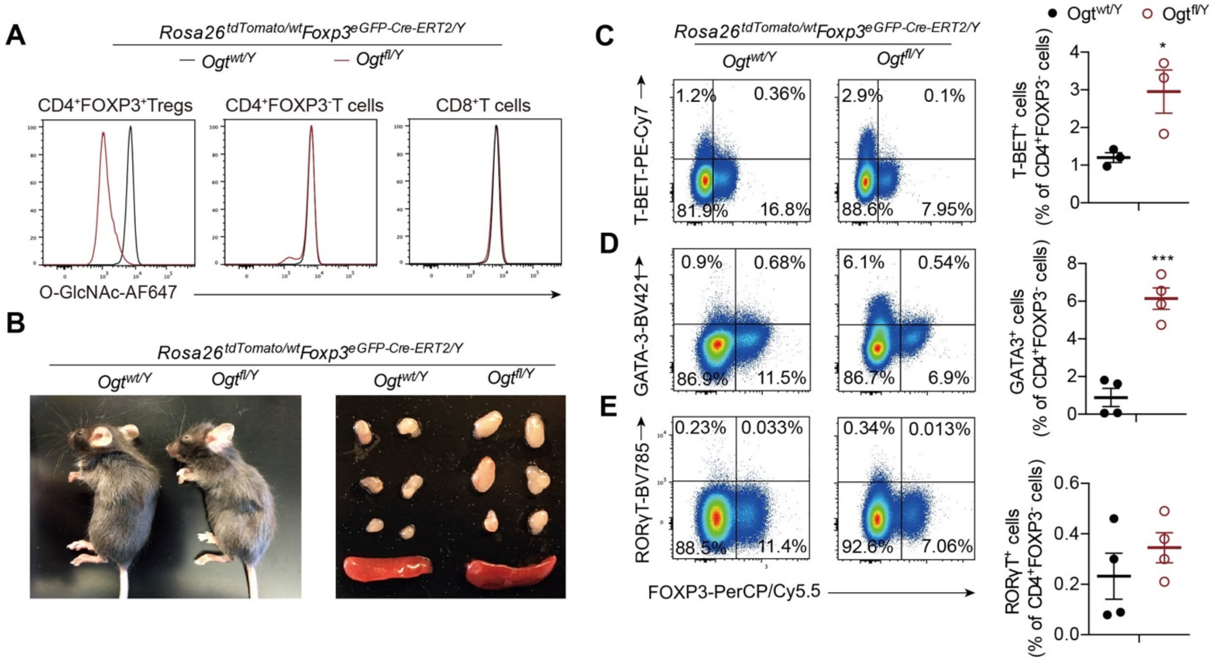

Supplementary Fig. 5 (A) MFI of O-GlcNAcylation in indicated cell populations from $Foxp3^{eGFP-Cre-ERT2/Y}$ $Ogt^{wt/Y}$ $Rosa26^{tdTomato/wt}$ and $Foxp3^{eGFP-Cre-ERT2/Y}$ $Ogt^{fl/Y}$ $Rosa26^{tdTomato/wt}$ male mice. (B) Representative images of 6-week-old $Foxp3^{eGFP-Cre-ERT2/Y}$ $Ogt^{wt/Y}$ $Rosa26^{tdTomato/wt}$ and $Foxp3^{eGFP-Cre-ERT2/Y}$ $Ogt^{fl/Y}$ $Rosa26^{tdTomato/wt}$ male mice fed tamoxifen food for 3 weeks and peripheral LNs and spleen. (C-E) T-BET$^+$ FOXP3$^-$ cells (C), GATA3$^+$ FOXP3$^-$ cells (D) and RORγT$^+$ FOXP3$^-$ cells (E) among CD4$^+$ T cells in LNs from $Foxp3^{eGFP-Cre-ERT2/Y}$ $Ogt^{wt/Y}$ $Rosa26^{tdTomato/wt}$ and $Foxp3^{eGFP-Cre-ERT2/Y}$ $Ogt^{fl/Y}$ $Rosa26^{tdTomato/wt}$ male mice, n = 3-4. Data are shown as mean ± s.e.m. *, $p<0.05$; ***, $p<0.001$ by unpaired student's *t*-test.

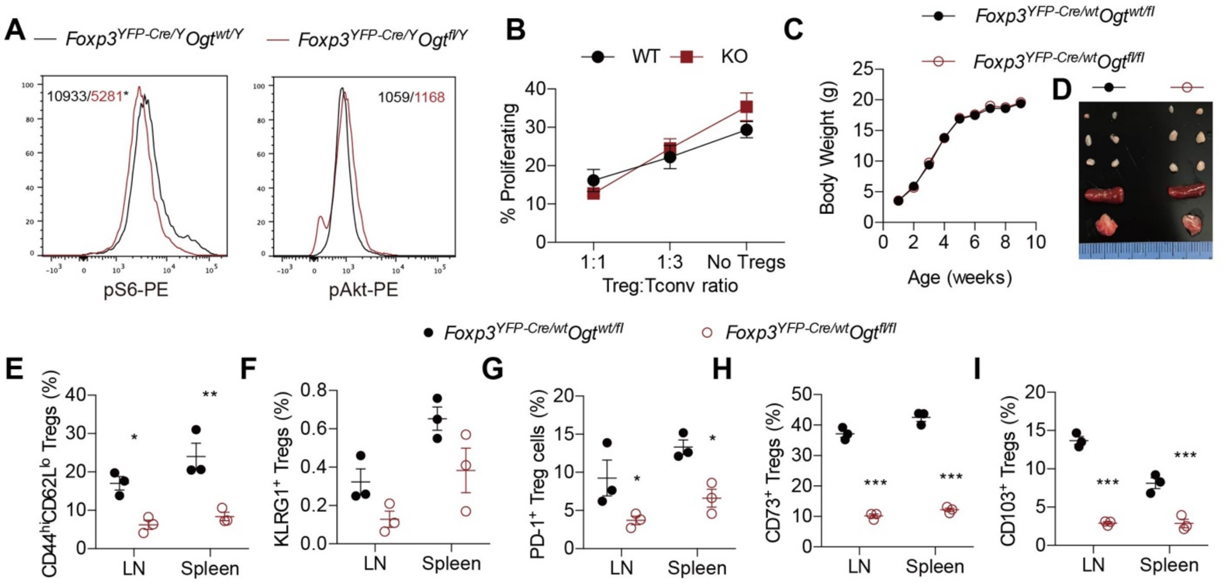

Supplementary Fig. 6 (A) MFI of pS6 and pS473-AKT among $CD4^{+}FOXP3^{+}$ Treg cells in LNs of 2-week-old *Foxp3$^{YFP\text{-}Cre/Y}$ Ogt$^{wt/Y}$* and *Foxp3$^{YFP\text{-}Cre/Y}$ Ogt$^{fl/Y}$* mice. (B) Suppression activity of OGT-sufficient and OGT-deficient Treg cells, the percentages of T conventional cell proliferation were measured by flow cytometry. (C, D) Body weight (C, n = 12) and representative images of peripheral LNs, spleen and thymus (D) of *Foxp3$^{YFP\text{-}Cre/wt}$Ogt$^{wt/fl}$* and *Foxp3$^{YFP\text{-}Cre/wt}$Ogt$^{fl/fl}$* female mice. (E-I) Frequencies of indicated cell populations among YFP^{+} Treg cells in the LNs and spleen from *Foxp3$^{YFP\text{-}Cre/wt}$ Ogt$^{wt/fl}$* and *Foxp3$^{YFP\text{-}Cre/wt}$ Ogt$^{fl/fl}$* female mice, n = 3 each group. Data are shown as mean ± s.e.m. *, $p<0.05$; **, $p<0.01$; ***, $p<0.001$ by unpaired student's t-test.

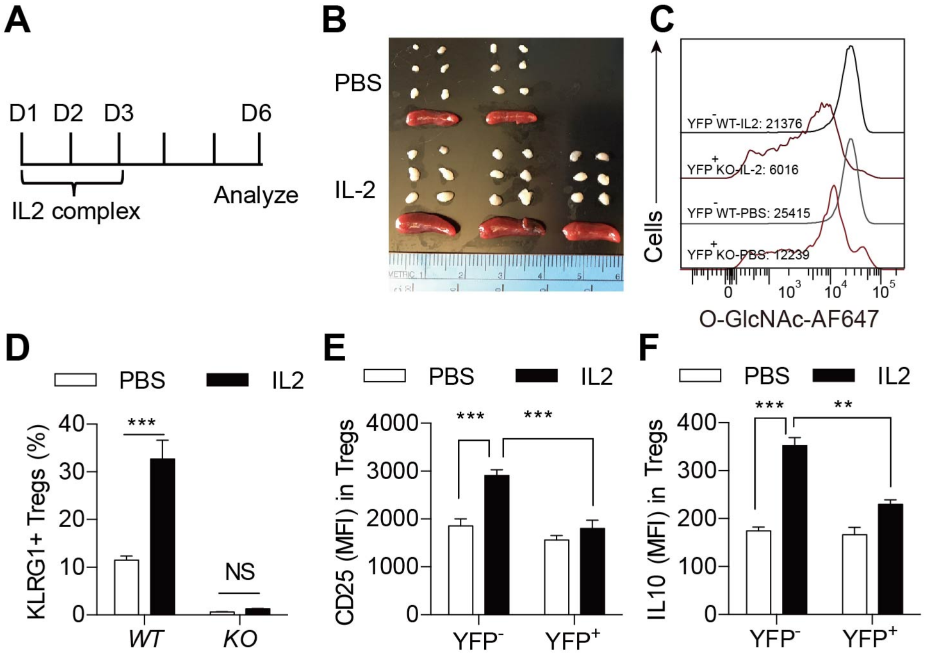

Supplementary Fig. 7 (A) Mice were injected with PBS or the IL-2 immune complex for 3 consecutive days and Treg cells were analyzed at day 6. (B, C) Representative images of peripheral LNs and Spleen(B) and MFI of O-GlcNAcylation among YFP^-OGT-sufficient or YFP^+OGT-deficient Treg cells ($CD4^+CD25^+GITR^+$) (C) from *Foxp3*$^{YFP\text{-}Cre/wt}$ *Ogt*$^{fl/fl}$ female mice injected with PBS or IL2 complex. (D) Frequencies of $CD44^+KLRG1^+$ cells among $CD4^+CD25^+FOXP3^+$ Treg cells in LNs and spleen from 2-week-old control and *Foxp3*$^{YFP\text{-}Cre/Y}$ *Ogt*$^{fl/Y}$ male mice injected with PBS or the IL-2 complex, n = 3. (E, F) MFI of CD25 (E) and IL10 (F) among YFP^-OGT-sufficient or YFP^+OGT-deficient Treg cells ($CD4^+CD25^+GITR^+$) from *Foxp3*$^{YFP\text{-}Cre/wt}$ *Ogt*$^{fl/fl}$ female mice injected with PBS or IL2 complex, n = 2-5. Data are shown as mean ± s.e.m. **, $p<0.01$; ***, $p<0.001$ by two-way ANOVA.

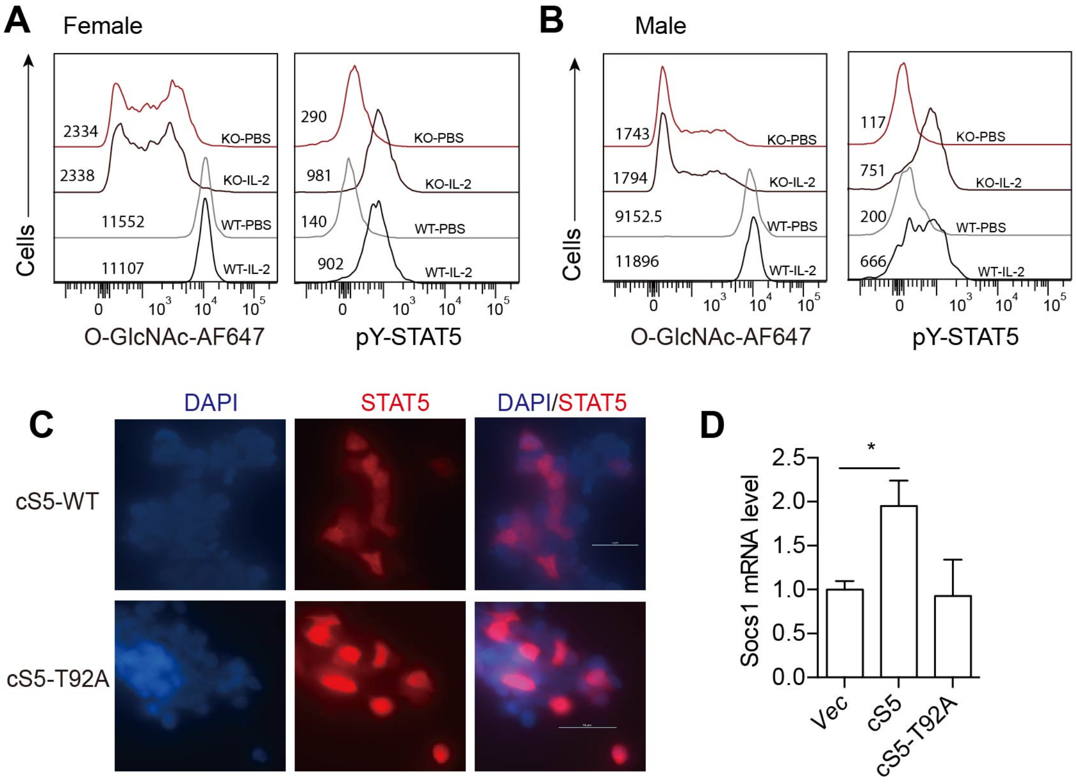

Supplementary Fig. 8 (A, B) MFI of O-GlcNAcylation and pY-STAT5 among YFP$^+$CD25$^+$ Treg cells in the LNs from *Foxp3$^{YFP\text{-}Cre/wt}$ Ogt$^{wt/fl}$* and *Foxp3$^{YFP\text{-}Cre/wt}$ Ogt$^{fl/fl}$* female mice (A) and *Foxp3$^{YFP\text{-}Cre/Y}$ Ogt$^{wt/Y}$* and *Foxp3$^{YFP\text{-}Cre/Y}$ Ogt$^{fl/Y}$* male mice (B) stimulated with IL-2 in vitro, PBS incubation was used as control. (C) Representative images of STAT5 nuclear translocation in HEK 293 cells transfected with cS5 or cS5-T92A. (D) mRNA levels of *Socs1* in OGT-deficient Treg cells after retrovirus infection as indicated, n = 3. Data are shown as mean ± s.e.m. *, $p<0.05$ by one-way ANOVA.

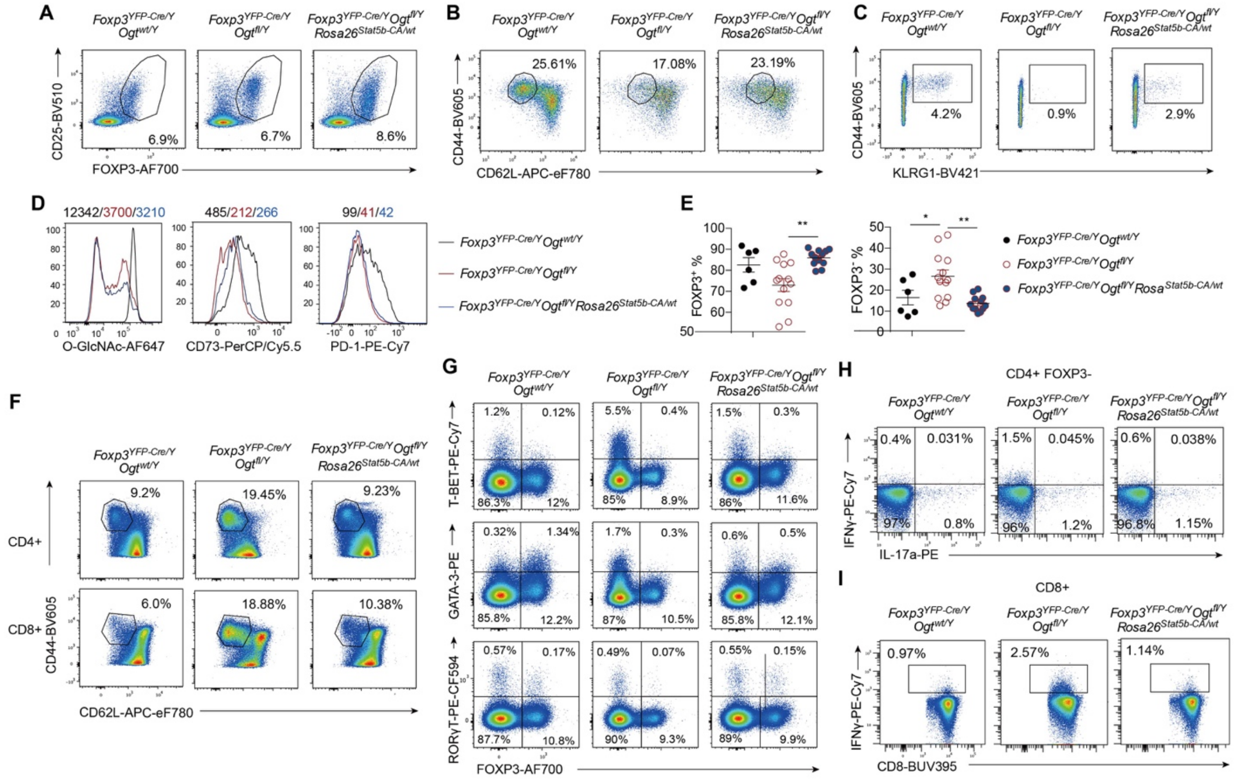

Supplementary Fig. 9 (A) Flow cytometry of FOXP3$^+$CD25$^+$ Treg cells among CD4$^+$ T cells in the LNs from *Foxp3$^{YFP-Cre/Y}$ Ogt$^{wt/Y}$*, *Foxp3$^{YFP-Cre/Y}$ Ogt$^{fl/Y}$* and *Foxp3$^{YFP-Cre/Y}$Ogt$^{fl/Y}$Rosa26$^{Stat5b-CA/wt}$* mice. (B, C) Flow cytometry of CD44^{hi}CD62L^{lo} eTreg cells (B) and CD44$^+$KLRG1$^+$ eTreg cells (C) among FOXP3$^+$CD25$^+$ Treg cells in the LNs from *Foxp3$^{YFP-Cre/Y}$ Ogt$^{wt/Y}$*, *Foxp3$^{YFP-Cre/Y}$ Ogt$^{fl/Y}$* and *Foxp3$^{YFP-Cre/Y}$Ogt$^{fl/Y}$Rosa26$^{Stat5b-CA/wt}$* mice. (D) MFI of O-GlcNAcylation, CD73 and PD-1 among FOXP3$^+$CD25$^+$ Treg cells. (E) Frequencies of FOXP3$^+$ and FOXP3$^-$ cells among CD4$^+$CD25$^+$YFP$^+$ Treg cells in the LNs, at least n = 6 each group. (F) Flow cytometry of CD44^{hi}CD62L^{lo} cells among CD4$^+$ and CD8$^+$ T cells in the LNs. (G) Flow cytometry of T-BET$^+$, GATA3$^+$ and RORγT$^+$ population among CD4$^+$FOXP3$^-$ cells in the LNs. (H, I) Flow cytometry of IFNγ-expressing cells among CD4$^+$FOXP3$^-$ (H) and CD8$^+$ T cells (I) in the LNs. Data are shown as mean ± s.e.m. *, $p<0.05$; **, $p<0.01$ by one-way ANOVA.

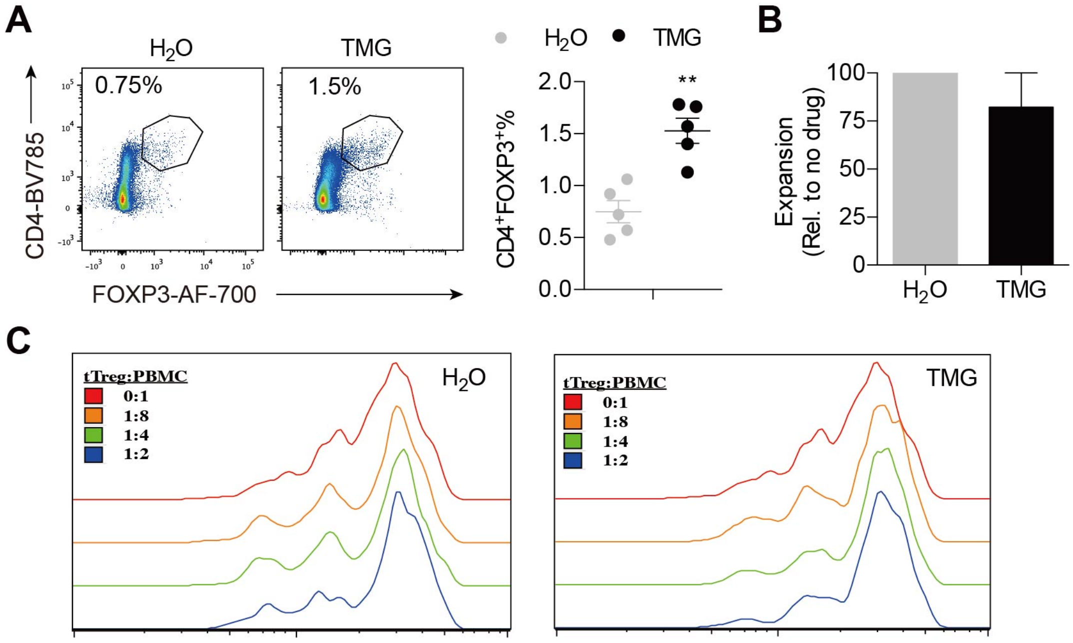

Supplementary Fig. 10 (A) Flow cytometry and quantification of the frequencies of mouse CD4$^+$FOXP3$^+$ Treg cells treated with TMG, H_2O treatment was used as a control, n = 5 each group. (B) Frequencies of human tTreg cells expansion treated with or without TMG for 7 days, n=3. (C) Representative plots showing the cell proliferation of CD8$^+$ cells labeled with CFSE. Data are shown as mean ± s.e.m. **, p<0.01 by unpaired student's t-test.

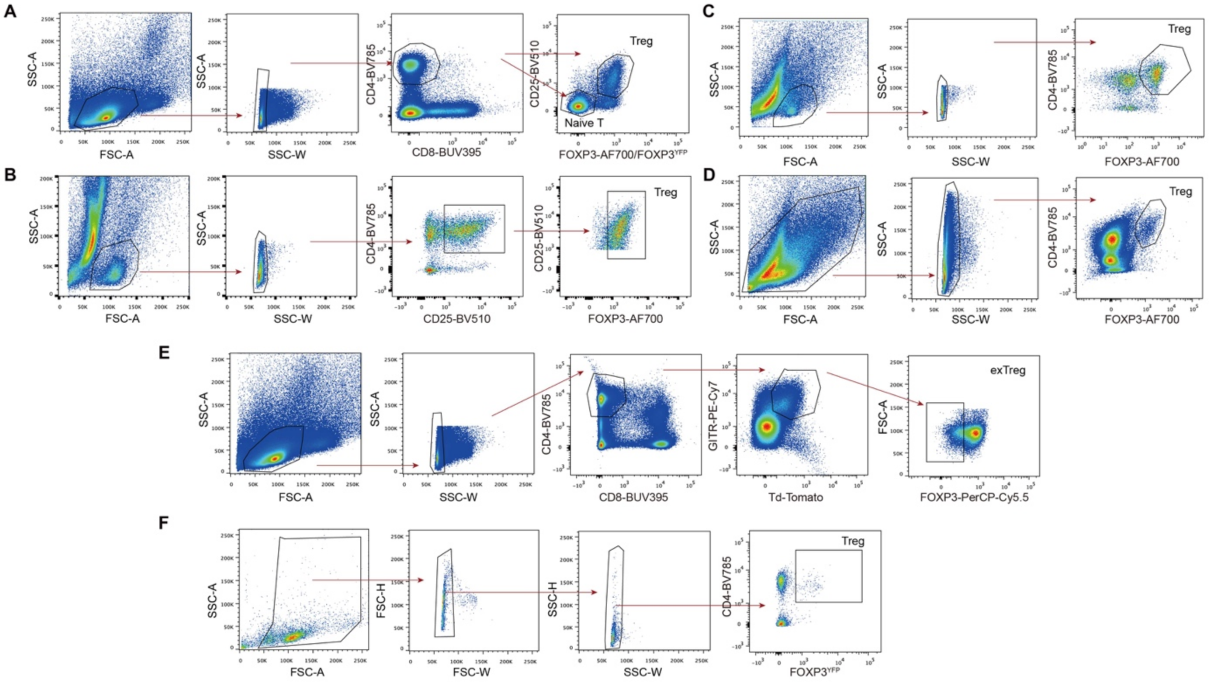

Supplementary Fig. 11 (A) Gating strategy to sort naïve T cells (CD4$^+$CD25$^-$FOXP$^-$) and Treg cells (CD4$^+$CD25$^+$FOXP3$^+$) from unpurified lymphocytes presented on Fig. 3 & Fig. 7. (B) Gating strategy to sort Treg cells (CD4$^+$CD25$^+$FOXP3$^+$) from wildtype mice treated with/without CD3/CD28 beads for in vitro purification and expansion presented on Fig. 1C. (C) Gating strategy to sort Treg cells (CD4$^+$FOXP3$^+$) from *Ubc-CreER*$^+$*Ogt*$^{fl/Y}$ and control mice treated with 4-OHT for in vitro purification and expansion presented on Fig. 2A-D or wildtype mice treated with/without TMG for in vitro purification and expansion presented on Fig. 8A-C. (D) Gating strategy to sort Treg cells (CD4$^+$FOXP3$^+$) from naïve T cells (CD4$^+$CD25$^-$) infected with retroviruses for in vitro purification and expansion presented on Fig. 2G, H. (E) Gating strategy to sort ex-Treg cells (Td-tomato$^+$GITR$^+$FOXP3$^-$) from *Foxp3*$^{eGFP-Cre-ERT2/Y}$ *Ogt*$^{wt/Y}$ *Rosa26*$^{tdTomato/wt}$ and *Foxp3* $^{eGFP-Cre-ERT2/Y}$ *Ogt*$^{fl/Y}$ *Rosa26*$^{tdTomato/wt}$ mice fed tamoxifen food presented in Fig. 4G-I. (F) Gating strategy to sort Treg cells (CD4$^+$FOXP3^{YFP+}) for RNA-seq presented in Fig. 6A.

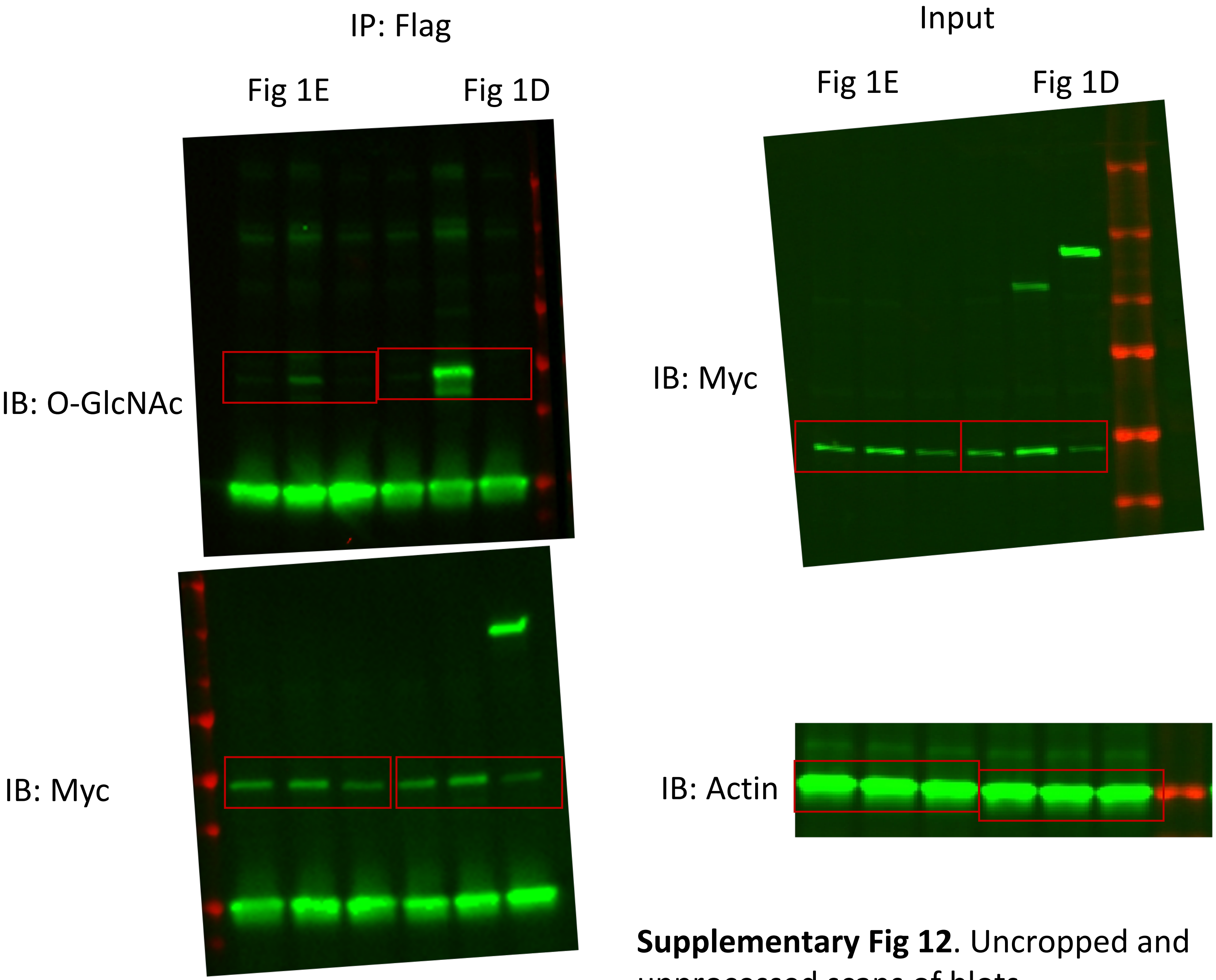

Supplementary Fig 12. Uncropped and unprocessed scans of blots

Fig 1F

1st day for Myc incubation

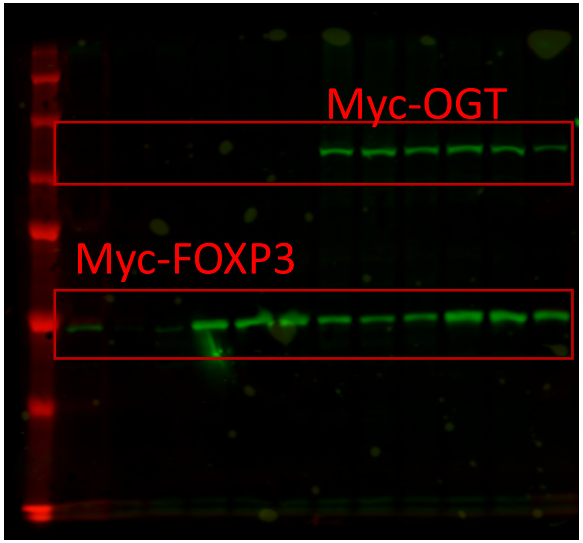

2nd day same membrane for Actin incubation

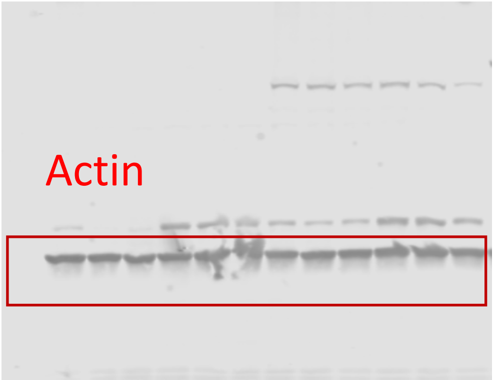

Supplementary Fig 12. Uncropped and unprocessed scans of blots

Fig 1G

1st day for Myc incubation

2nd day same membrane for Actin incubation

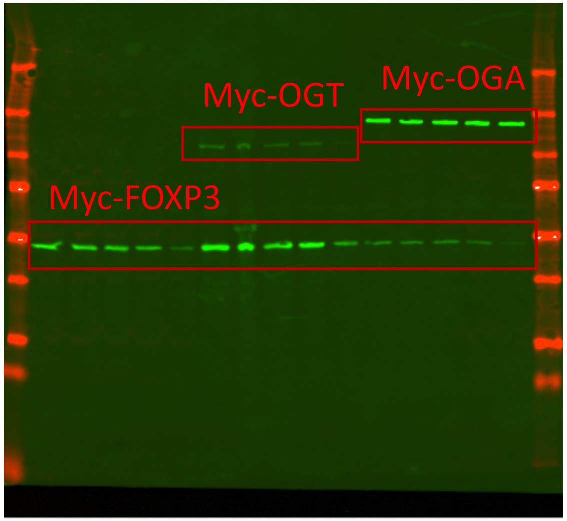

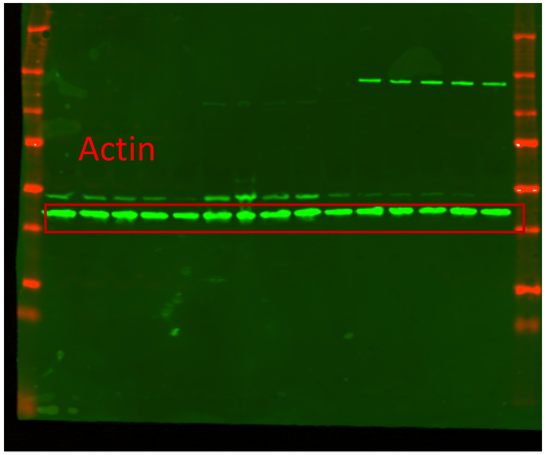

Supplementary Fig 12. Uncropped and unprocessed scans of blots

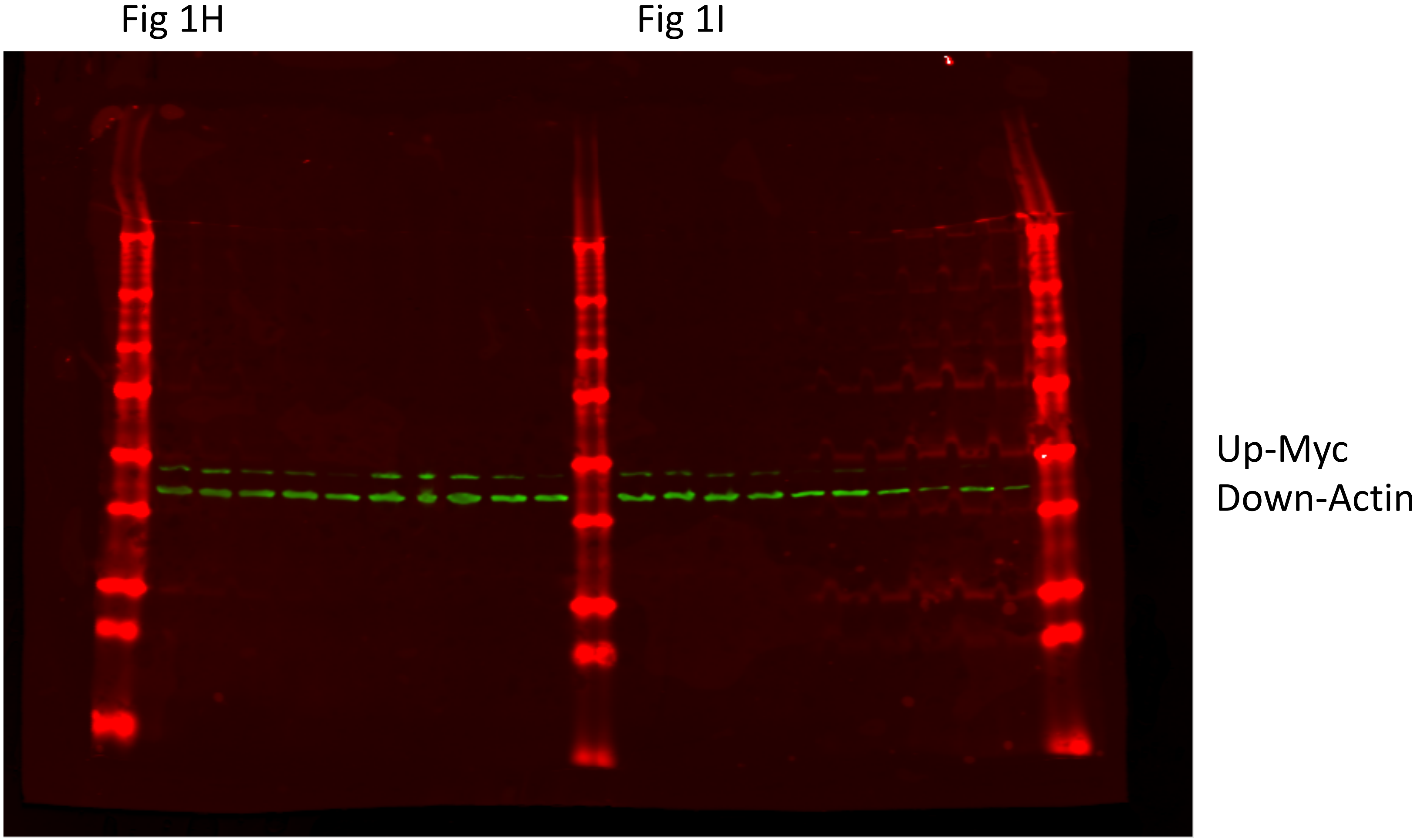

Supplementary Fig 12. Uncropped and unprocessed scans of blots

Fig 2E

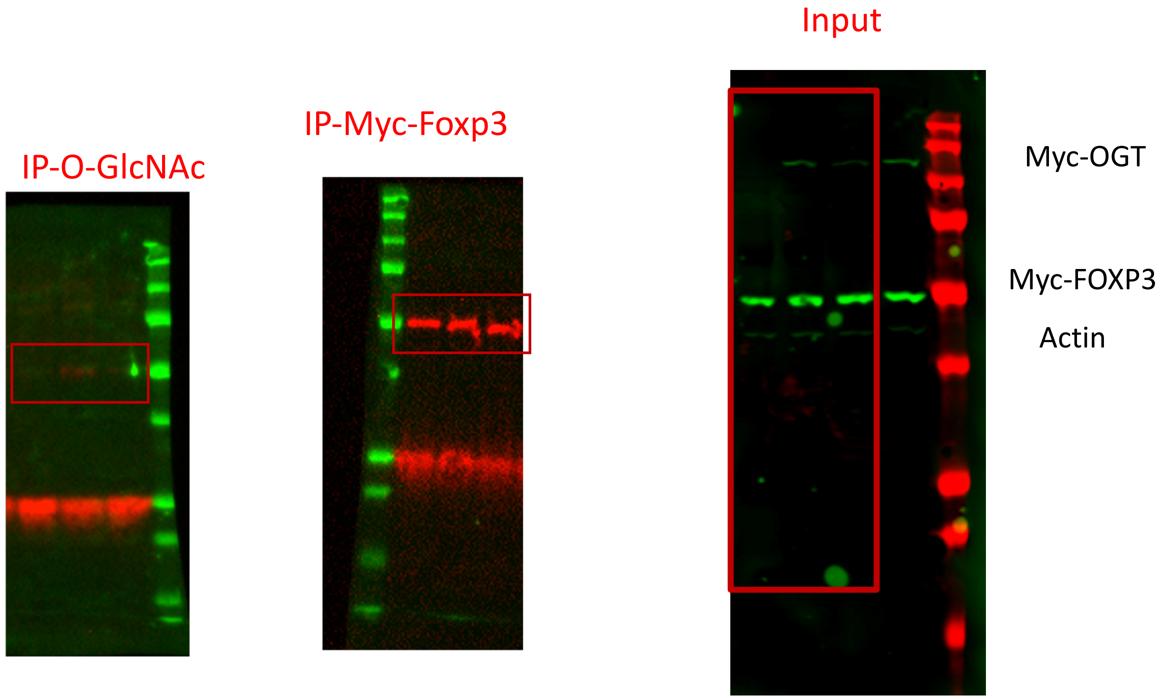

Supplementary Fig 12. Uncropped and unprocessed scans of blots

Fig 2F

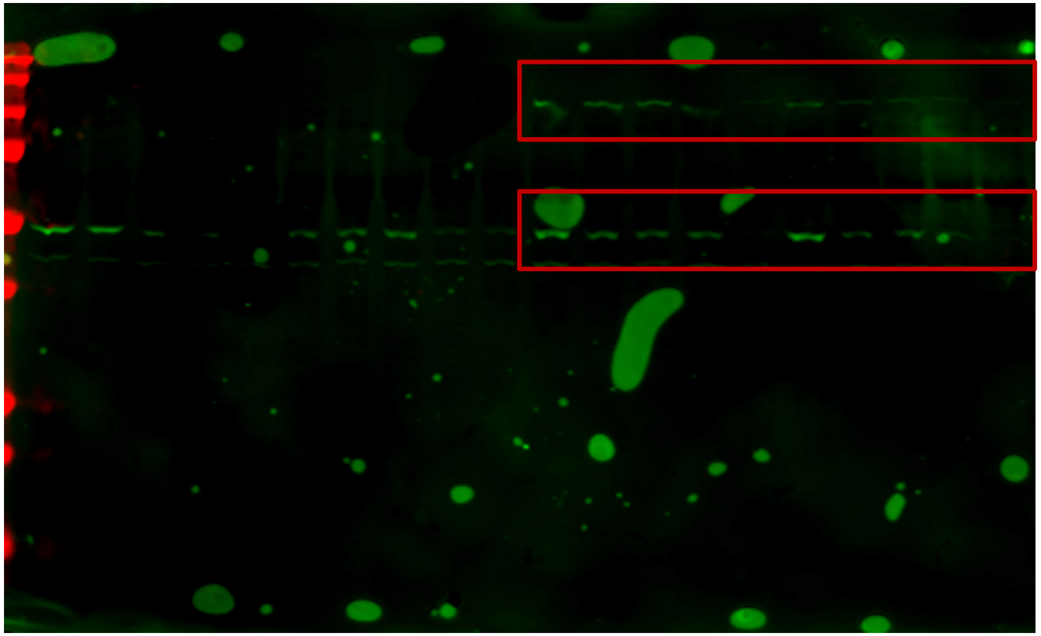

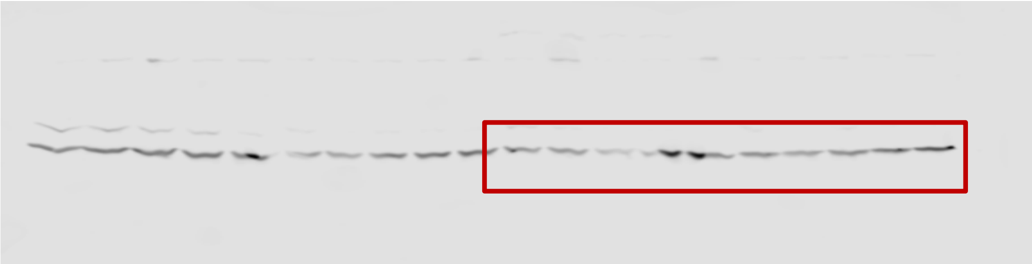

Supplementary Fig 12. Uncropped and unprocessed scans of blots

Supplementary Fig 1B

O-GlcNAc

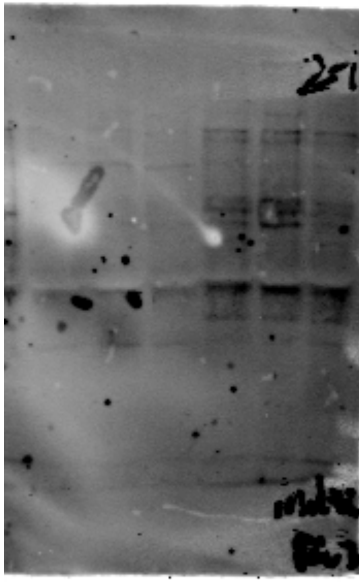

Actin

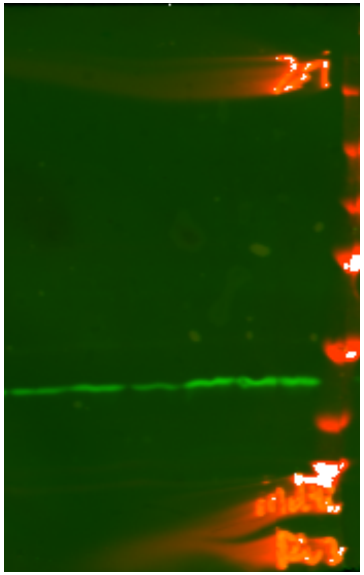

Supplementary Fig 12. Uncropped and unprocessed scans of blots

Supplementary Fig 1C

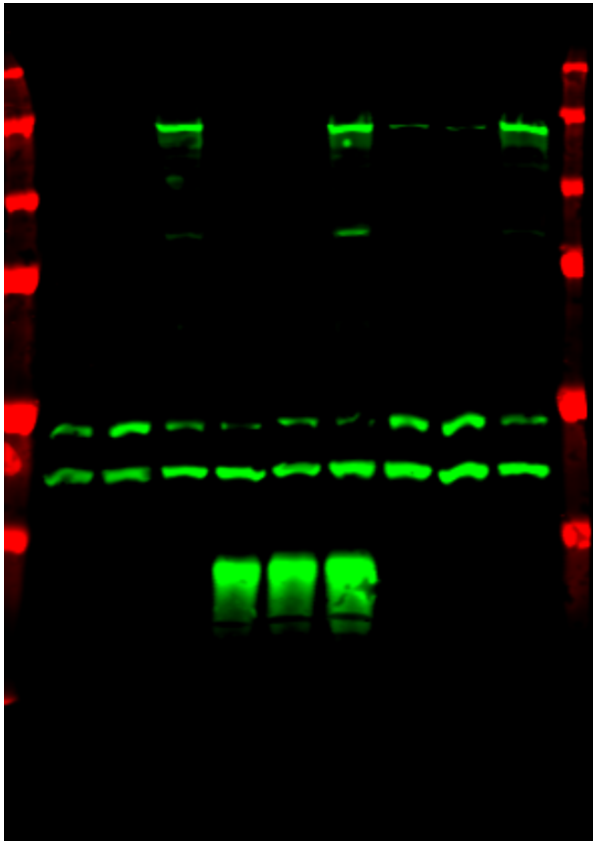

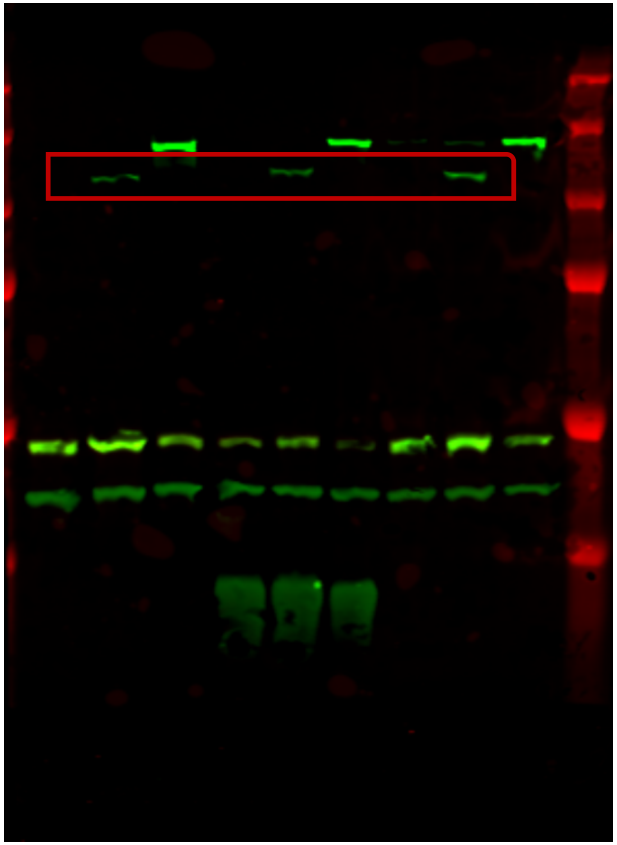

Supplementary Fig 12. Uncropped and unprocessed scans of blots

Supplementary Fig 4G

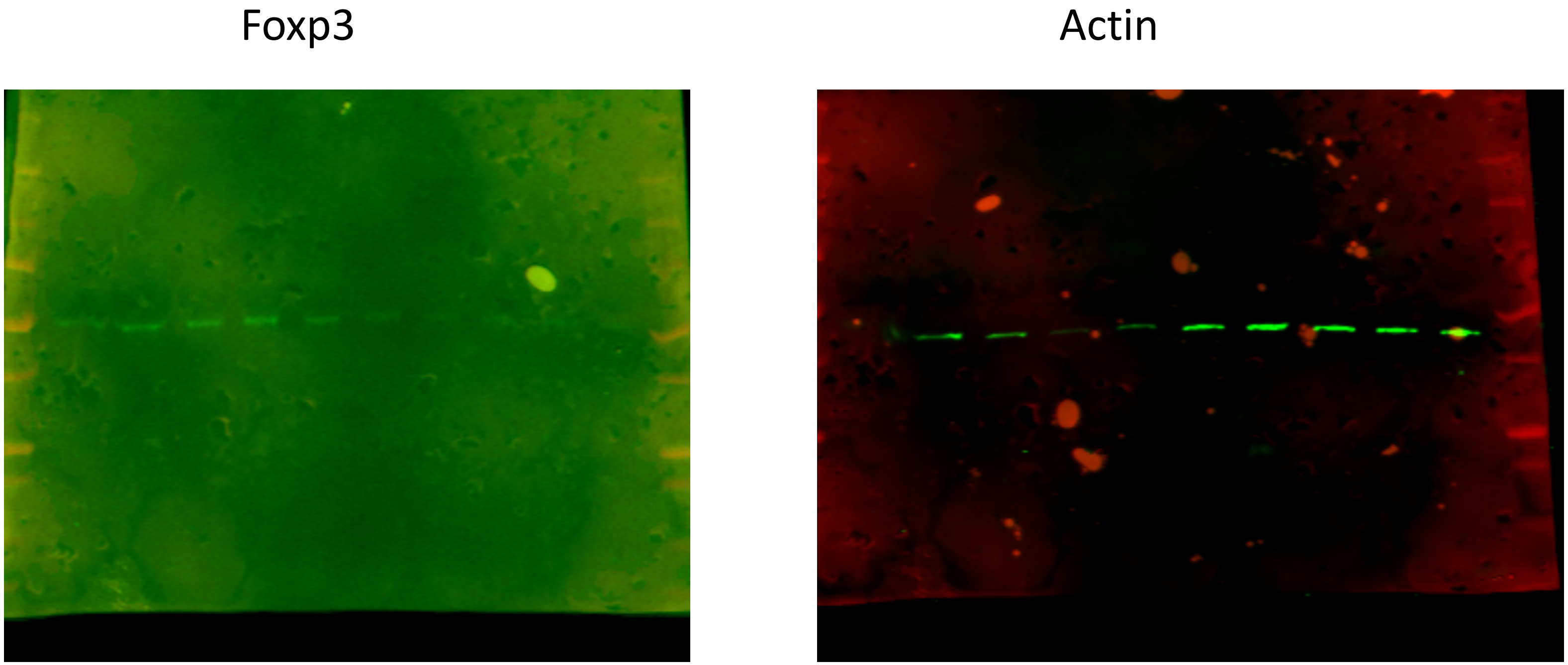

Supplementary Fig 12. Uncropped and unprocessed scans of blots

Supplementary Table 1

Primer name	Sequence
Human *SOCS1* sense	5’-CACGCACTTCCGCACATTC-3’
Human *SOCS1* antisense	5’-TAAGGGCGAAAAAGCAGTTCC-3’
Human *SOCS 3* sense	5’-CCTGCGCCTCAAGACCTTC-3’
Human *SOCS 3* antisense	5’-GTCACTGCGCTCCAGTAGAA-3’
Human *18S* sense	5’-GCTGGAGGACTCATGTTCAAC -3’
Human *18S* antisense	5’-CCTTGGGTCAAGTTCACAAGC-3’
Mouse *Klrg1* sense	5’-TTTGGGGCTTTTGACTGTGAT-3’
Mouse *Klrg1* antisense	5’-TGTAAGGAGATGTGAGCCTTTGT-3’
Mouse *Pd-1* sense	5’-ACCCTGGTCATTCACTTGGG-3’
Mouse *Pd-1* antisense	5’-CATTTGCTCCCTCTGACACTG-3’
Mouse *Cd44* sense	5’-TCGATTTGAATGTAACCTGCCG-3’
Mouse *Cd44* antisense	5’-CAGTCCGGGAGATACTGTAGC-3’
Mouse *Cd73* sense	5’-GGACATTTGACCTCGTCCAAT-3’
Mouse *Cd73* antisense	5’-GGGCACTCGACACTTGGTG-3’
Mouse *Socs1* sense	5’-CTGCGGCTTCTATTGGGGAC-3’
Mouse *Socs1* antisense	5’-AAAAGGCAGTCGAAGGTCTCG-3’
Mouse *Socs3* sense	5’-ATGGTCACCCACAGCAAGTTT-3’
Mouse *Socs3* antisense	5’-TCCAGTAGAATCCGCTCTCCT-3’
Mouse *Ogt* sense	5’-AAGAGGCACGCATTTTTGAC-3’
Mouse *Ogt* antisense	5’-ATGGGGTTGCAGTTCGATAG-3’
Mouse *18s* sense	5’-ACCGCAGCTAGGAATAATGGA-3’
Mouse *18s* antisense	5’-GCCTCAGTTCCGAAAACCA-3’

Supplementary Table 2

Name	Vendor	Catalog no.	Dilution
CD4	BioLegend	100551	1:200
CD4	BioLegend	100548	1:200
CD4	eBioscience	48-0042-82	1:200
CD25	BioLegend	102041	1:200
CD8	BD Biosciences	563786	1:200
CD8	BD Biosciences	563068	1:200
PD-1	eBioscience	25-9985-80	1:200
PD-1	BioLegend	135208	1:200
CD44	BioLegend	103047	1:200
CD44	BioLegend	103012	1:200
CD62L	eBioscience	47-0621-80	1:200
KLRG1	BD Horizon	562897	1:200
Nrp-1	BioLegend	145209	1:200
TCRβ chain	BD pharmingen	560705	1:200
CD73	BioLegend	127214	1:200
CD103	BioLegend	121406	1:200
CD103	BioLegend	121415	1:200
Helios	BioLegend	137222	1:200
GITR	Thermo Fisher	25-5874-82	1:200
NK-1.1	BioLegend	108708	1:200
CD19	Tonbo Biosciences	65-0193-U100	1:200
CD45R/B220	BD Biosciences	563793	1:200
FOXP3	eBioscience	56-7773-82	1:50
O-GlcNAc	Abcam	201994	1:400
Ki-67	eBioscience	12-5698-82	1:100
T-bet	BioLegend	644824	1:100
GATA-3	eBioscience	12-9966-42	1:100
GATA-3	BD Horizon Biosciences	563349	1:20
RORγT	BD Horizon	562684	1:100
RORγT	BD Horizon	564723	1:100
GFP	Thermo Fisher	A-21311	1:400
Annexin V	BD Biosciences	550474	1:100
STAT5 (pY694)	BD Biosciences	612516	1:100
IFNγ	TONBO biosciences	60-7311-U100	1:100
IL4	BioLegend	504105	1:100
IL17A	BD Biosciences	559502	1:100
IL10	BD Biosciences Pharmingen	554467	1:100
IL5	Caltag Laboratories	RM9064	1:100
IL13	Invitrogen	47-7133-80	1:100
BLIMP-1	BioLegend	150007	1:100
pAKT (Ser473)	Cell Signaling Technology	#4060	1:100
pS6(Ser235, 236)	eBioscience	12-9007-41	1:100
rabbit IgG (H+L)	Life Technologies	A21429	1:100
GZMB	eBioscience	12-8899-71	1:100
