# Supplementary material for: The lineage stability and suppressive program of regulatory T cells require protein O-GlcNAcylation
Source: Nat Commun. 2019 Jan 21;10:354. doi: 10.1038/s41467-019-08300-3 (PMC6341091; doi:10.1038/s41467-019-08300-3)
Supplement: Supplementary file 3 — Description of Additional Supplementary Files [file 41467_2019_8300_MOESM3_ESM.pdf]

Description of Additional Supplementary Files

Supplementary Data 1:

Identification of FOXP3 O-GlcNAcylation sites. Top, sequence, location, and HexNAc sites of O-GlcNAcylated peptides identified by Mass Spectrometry. Bottom, full sequence of the purified mouse FOXP3 protein used for Mass Spectrometry, with annotations on tags and covered regions.
